# Active Vaccination With EMMPRIN-Derived Multiple Antigenic Peptide (161-MAP) Reduces Angiogenesis in a Dextran Sodium Sulfate (DSS)-Induced Colitis Model

**DOI:** 10.3389/fimmu.2018.02919

**Published:** 2018-12-10

**Authors:** Elina Simanovich, Vera Brod, Michal A. Rahat

**Affiliations:** ^1^Immunotherapy Laboratory, Carmel Medical Center, Haifa, Israel; ^2^The Ruth and Bruce Rappaport Faculty of Medicine, Technion-Israel Institute of Technology, Haifa, Israel

**Keywords:** angiogenesis, multiple antigenic peptide (MAP), active peptide vaccination, DSS-induced colitis, EMMPRIN/CD147

## Abstract

Ulcerative colitis (UC) is an autoimmune disease that affects the colon and shares many clinical and histological features with the dextran sulfate sodium (DSS)-induced colitis model in mice. Angiogenesis is a critical component in many autoimmune diseases, as well as in the DSS-induced colitis model, and is it partially mediated by EMMPRIN, a multifunctional protein that can induce the expression of both the potent pro-angiogenic vascular endothelial growth factor (VEGF) and matrix metalloproteinases (MMPs). We asked whether targeting EMMPRIN by active vaccination, using a novel, specific epitope in the protein, synthesized as a multiple antigenic peptide (MAP), could trigger beneficial effects in the DSS-induced colitic C57BL/6J mice. Mice were vaccinated with four boost injections (50 μg each) of either 161-MAP coding for the EMMPRIN epitope or the scrambled control peptide (Scr-MAP) emulsified in Freund's adjuvant. We show that male mice that were vaccinated with 161-MAP lost less weight, demonstrated improved disease activity indices (DAI), had reduced colitis histological score, and their colons were longer in comparison to mice vaccinated with the Scr-MAP. The 161-MAP vaccination also reduced serum and colon levels of EMMPRIN, colon concentrations of VEGF, MMP-9, and TGFβ, and vessel density assessed by CD31 staining. A similar effect was observed in female mice vaccinated with 161-MAP, including weight loss, colitis histological score, colon length, colon levels of EMMPRIN and colon concentrations of VEGF. However, for female mice, the changes in DAI values, EMMPRIN serum levels, and MMP-9 and TGFβ colon concentrations did not reach significance. We conclude that our strategy of alleviating autoimmunity in this model through targeting angiogenesis by actively vaccinating against EMMPRIN was successful and efficient in reducing angiogenesis.

## Introduction

Inflammatory bowel disease (IBD) is a group of chronic inflammatory diseases, with the two major diseases being Crohn's disease (CD) and ulcerative colitis (UC). UC is believed to be an autoimmune disease that primarily affects the large intestine, with unknown etiology. However, a local increase in the concentrations of reactive oxygen species (ROS) and pro-inflammatory cytokines (primarily, TNFα, IL-1β, and IL-17), was identified in UC patients, and these were speculated to increase the risk of colorectal cancer in chronic inflammation (1–3).

The DSS-induced colitis model used in rats and mice is widely used as an experimental model of IBD that demonstrates clinical and histopathological features similar to the human autoimmune UC (3, 4). DSS is thought to induce a chemical injury in intestinal epithelial cells, which causes them to lose barrier functions and consequently exposes the Lamina Propria (LP) to antigens and intestinal bacteria that enhance inflammation (1). Different concentrations of DSS, usually ranging between 1 and 3%, have been used to achieve moderate, mild or severe intestinal injury, varying time of healing and repair accordingly (5). Similar to UC, the pro-inflammatory response and increased reactive oxygen species (ROS) are implicated in the continued tissue damage caused in DSS-induced colitis (2).

Angiogenesis has been linked to chronic inflammation, and has been shown to be a critical component of the pathogenesis of DSS-induced colitis and is associated with disease severity, as it promotes leukocyte influx and supplies the necessary oxygen and nutrients to the inflamed tissue (6). VEGF is a known potent pro-angiogenic factor that links angiogenesis and inflammation by promoting endothelial cell proliferation, migration, tube formation and vascular permeability, as well as increasing neutrophil adhesion through the activation of NF-κB and increased expression of adhesion molecules (7, 8). Matrix metalloproteinases (MMPs) can remodel the ECM to facilitate endothelial cell migration, release VEGF that is bound to the ECM, or conversely, degrade collagen XVIII to produce the anti-angiogenic factor endostatin (9). Thus, both VEGF and MMPs, particularly MMP-9, promote angiogenesis. Increased permeability, characteristic of angiogenic vessels, further contributes to UC progression, as it reduces barrier functions and allows interaction of lumen bacteria with LP immune cells (10).

EMMPRIN (also called CD147 or basigin) is a transmembranal protein with multiple functions. Depending on the protein it binds to, EMMPRIN can be involved in cell metabolism when it chaperones the monocarboxylate transporters MCT-1 and MCT-4, it can serve as a leukocyte chemoattractant when it binds to extracellular cyclophilin A/B, and it becomes an adhesion molecule when it binds to integrins and to E-selectin, to name just a few functions (11–13). However, its most familiar activity is mediated through homophilic interactions of membranal-soluble or membranal-membranal EMMPRIN molecules (14), which induce the expression of VEGF and several types of MMPs, rendering EMMPRIN an important pro-angiogenic factor (15–18).

We have recently identified a novel epitope in the EMMPRIN protein extracellular domain I, which is specifically responsible for the induction of both VEGF and MMP-9 (19). We have synthesized this epitope as an octa-branched multiple antigenic peptide (designated 161-MAP), and used it either in a therapeutic or a prophylactic manner to vaccinate mice that were implanted with the CT26 colon carcinoma tumors subcutaneously, or that were intravenously injected with this cell line to generate an experimental metastasis model. Vaccination against EMMPRIN resulted in the inhibition, and even regression of both tumors and metastases, partly through the reduction in vessel density, and through reduced expression of EMMPRIN, VEGF and MMP-9, cumulatively decreasing angiogenesis. In view of the importance of angiogenesis in colitis, we now ask whether the same vaccination against EMMPRIN could also affect chronic inflammation in a mouse DSS-induced colitis model.

## Results

### 161-MAP Active Vaccination Ameliorates Disease Severity in DSS-Induced Colitis

**Figure 1:**
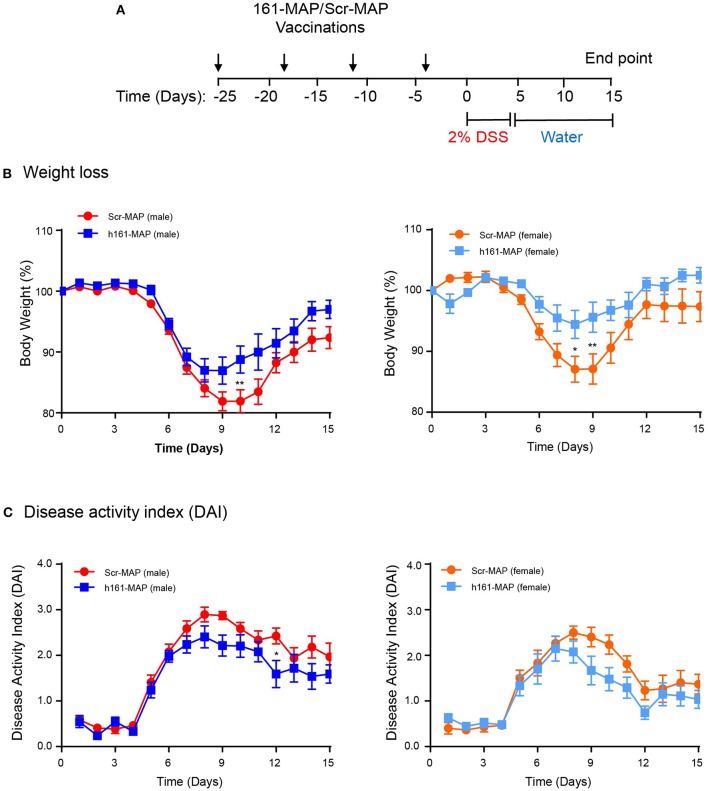
161-MAP active vaccination improves disease severity. **(A)** The experimental design is shown. Male and female C57Bl/6J mice were randomly assigned to the Scr-MAP vaccinated control group, or to the 161-MAP vaccinated experimental group. Mice received four vaccine boost injections every 7 days (arrows) prior to administration of 2% DSS in their drinking water for 5 consecutive days (marked). For the remaining time the mice received regular water, and after 10 days they were euthanized. After receiving DSS, mice were monitored daily and their **(B)** loss of weight or **(C)** the disease activity index (DAI) were observed (*n* = 12 for male mice, *n* = 9 for female mice). ^*^p < 0.05, ^**^p < 0.01 relative to the Scr-MAP vaccinated mice at the same day.

We have chosen to vaccinate the mice with Scr-MAP or 161-MAP in a prophylactic manner, before the induction of colitis by DSS, to allow the adaptive immunity to prepare fully to the DSS challenge. Four days after the last vaccine injection the mice were supplied with DSS dissolved in their drinking water for 5 consecutive days, and 15 days after the introduction of DSS we euthanized the mice. The design of the experiment is shown (Figure 1A). DSS induced colonic damage that was manifested both by weight loss and by the disease activity index (DAI), which was calculated by factoring in weight loss, diarrhea, and occult blood or rectal bleeding. In the Scr-MAP vaccinated control mice, weight loss and DAI gradually increased and peaked around day 9 both in male and female mice. Following this peak, mice exhibited reduction in the change of weight and in the DAI (Figure 1B), suggesting that repair of the colonic damage had begun. A similar trend was observed for the weight loss of the161-MAP vaccinated mice, but although the peak was evident on the same days as the control group, it was reduced in comparison (by 1.65-fold, *p* < 0.01 for the male mice, by 2.9-fold, *p* < 0.05 for the female mice). Disease severity, assessed by the DAI, showed significant results only for male mice. It gradually elevated and peaked around day 9, and then moderately declined in both Scr-MAP and 161-MAP vaccinated mice, but not back to the levels prior to DSS administration. However, in days 9 through 12, the disease in the control group was more severe (by 23 and 35%, *p* < 0.05, Figure 1C). This observation suggests that damage and inflammation are still preset 10 days after mice were no longer exposed to DSS, and that the 161-MAP active vaccination exerted a protective influence. It is also noteworthy that starting on day 10, the Scr-MAP vaccinated females demonstrated significantly reduced change in weight (*p* < 0.001) and DAI scores (*p* < 0.01), in comparison to the Scr-MAP vaccinated males, suggesting that the females exhibited reduced inflammation.

**Figure 2:**
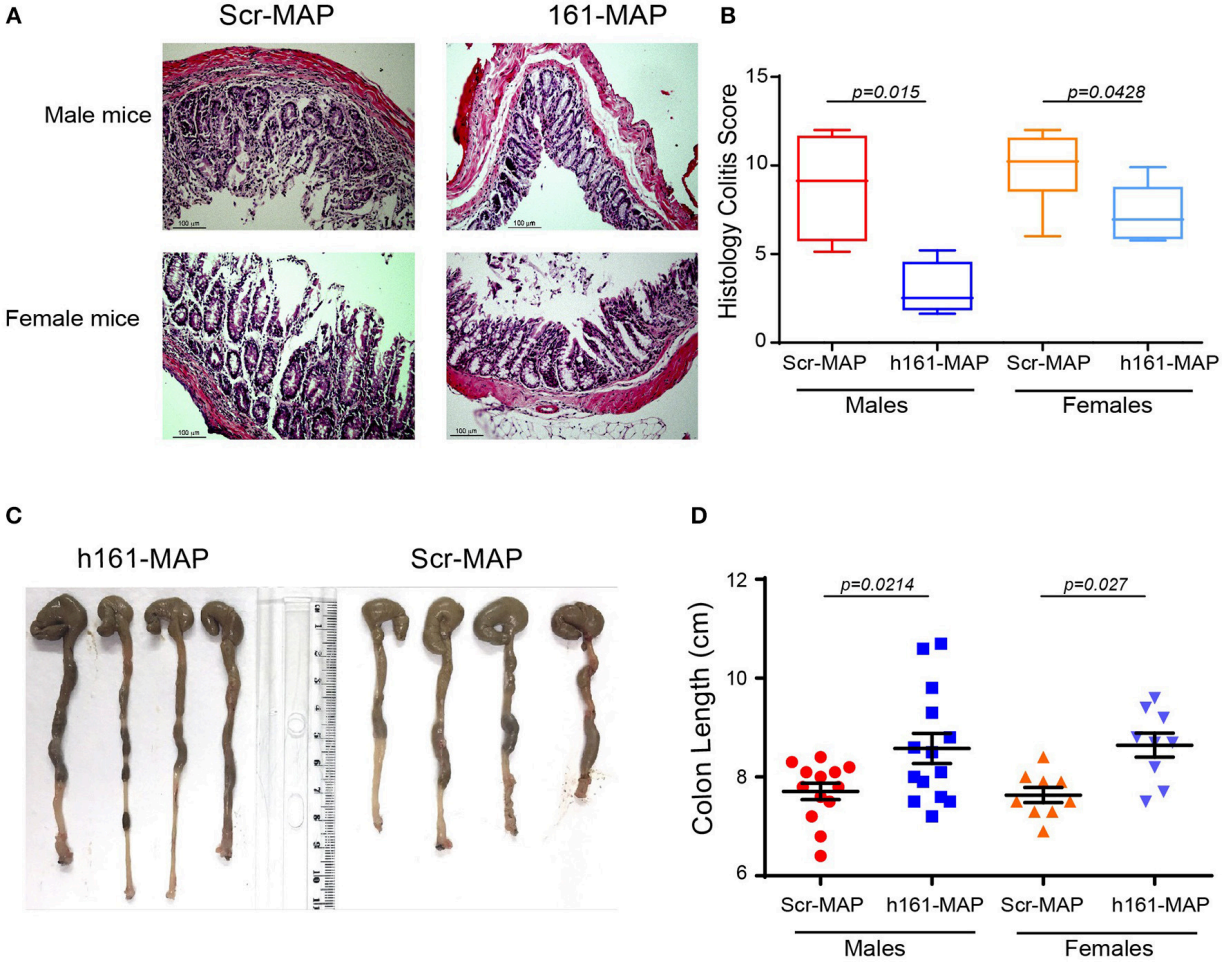
161-MAP active vaccination prevents damage and extends colon length. Fifteen days after the beginning of DSS-treatment, mice were euthanized and their colons were harvested, formalin-fixed and paraffin-embedded. Colon tissue sections were H&E stained and analyzed for their histological scores. **(A)** Representative images and **(B)** the assessment of the histological score (*n* = 6 in each group). Scale bar is 100 μm. **(C)** Representative images of the entire colon removed from male mice, and **(D)** measurement of the colon lengths (*n* = 12 for the male groups, *n* = 9 for the female groups).

Histological analysis of the colon in the control groups revealed damage to the epithelial layer, crypt loss and destruction, and increased leukocyte infiltration to the lamina propria (LP) and to the submucosa (Figures 2A,B). In comparison, in the 161-MAP vaccinated groups, crypt structures and epithelial lining were less damaged, and the immune infiltrate was reduced, as was reflected in the histological scores (Figure 2B, 95% CI for males [1.6, 10.15], 95% CI for females [0.1, 5.02]). Additionally, colon length, where shortening is a marker of inflammation, was increased in the 161-MAP vaccinated mice (by 12% in both male and female mice, *p* < 0.05, 95% CI for males [0.14, 1.6], and for females [0.4, 1.6], Figures 2C,D).

### 161-MAP Active Vaccination Reduces Angiogenesis

**Figure 3:**
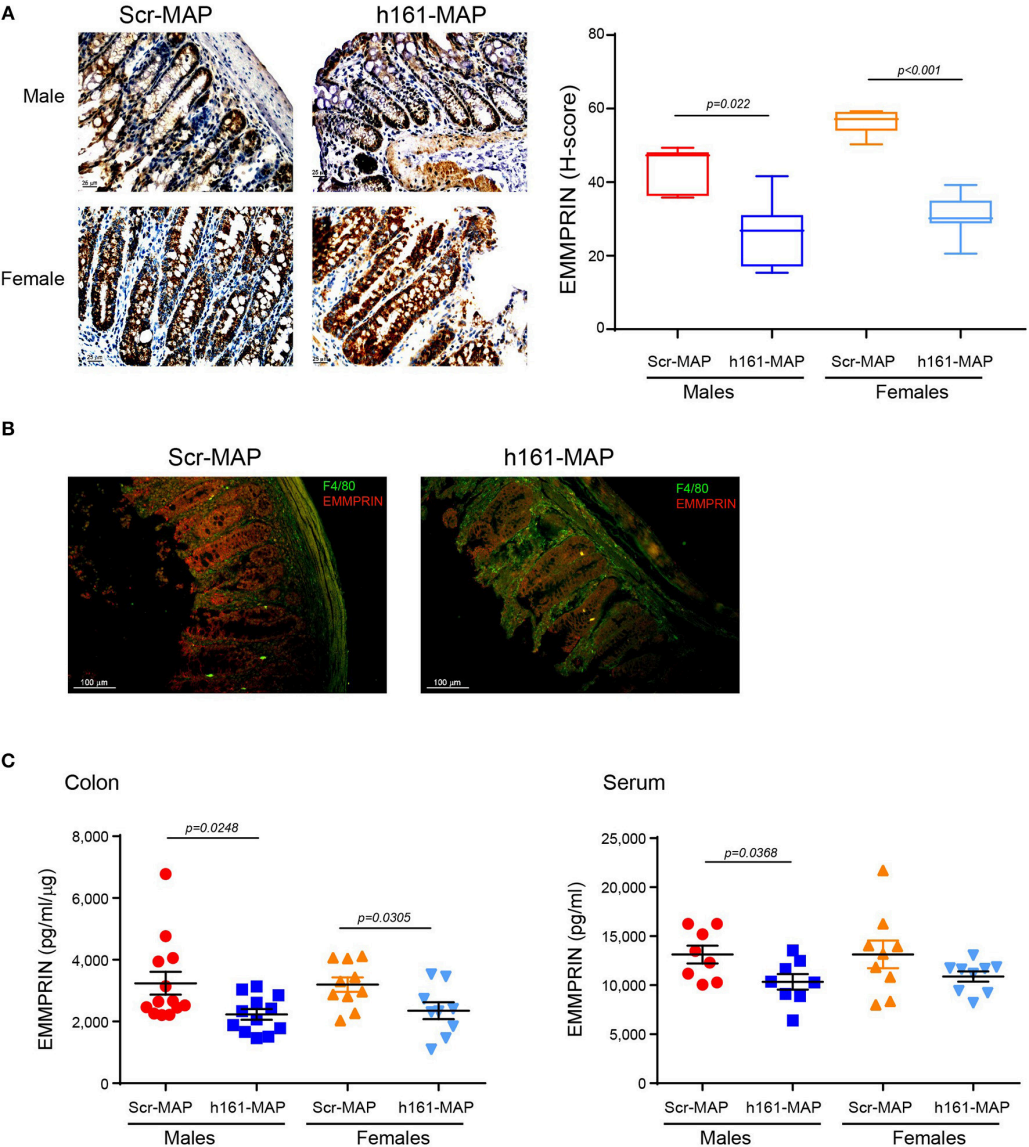
161-MAP active vaccination reduces EMMPRIN expression in the colon. **(A)** Representative images of colon sections stained for EMMPRIN and their quantification using the H-score (*n* = 5 per group). Scale bar is 25 μm **(B)** Representative image of fluorescently labeled EMMPRIN (red) and macrophages (green). Scale bar is 100 μm. **(C)** Determination of EMMPRIN concentrations in colon lysates (*n* = 9–10 per group), and in serum samples (*n* = 8–9 per group) by ELISA.

To examine whether the 161-MAP active vaccination targeted the EMMPRIN protein in the colon, we stained for the protein in the colon tissue sections. EMMPRIN is highly expressed in the colon, and therefore, staining in both cases was strong. However, the immune reactive score (H-score), which takes into account both the intensity of the staining and the number of cells stained with each intensity, allowed us to observe a reduction in EMMPRIN expression in the 161-MAP vaccinated group (Figure 3A), in both male (by 1.6-fold, *p* = 0.0022, 95% CI [7.16, 25.43]) and female mice (by 1.8-fold, *p* < 0.0001, 95% CI [19.9, 30.45]). Moreover, reduced EMMPRIN expression is clearly visualized in the crypts' epithelial cells from 161-MAP vaccinated mice, whereas an increase in infiltrating macrophages is clearly seen in the LP (Figure 3B). Lastly, determination of EMMPRIN levels in the colon lysates (Figure 3C) showed a marked reduction in the 161-MAP vaccinated male (by 1.45-fold, *p* = 0.0248, 95% CI [140, 1878]) and female (by 1.36-fold, *p* = 0.03, 95% CI [89.1, 1596]) mice, whereas EMMPRIN levels in the circulation were markedly reduced only in the male group (by 1.3-fold, *p* = 0.037).

**Figure 4:**
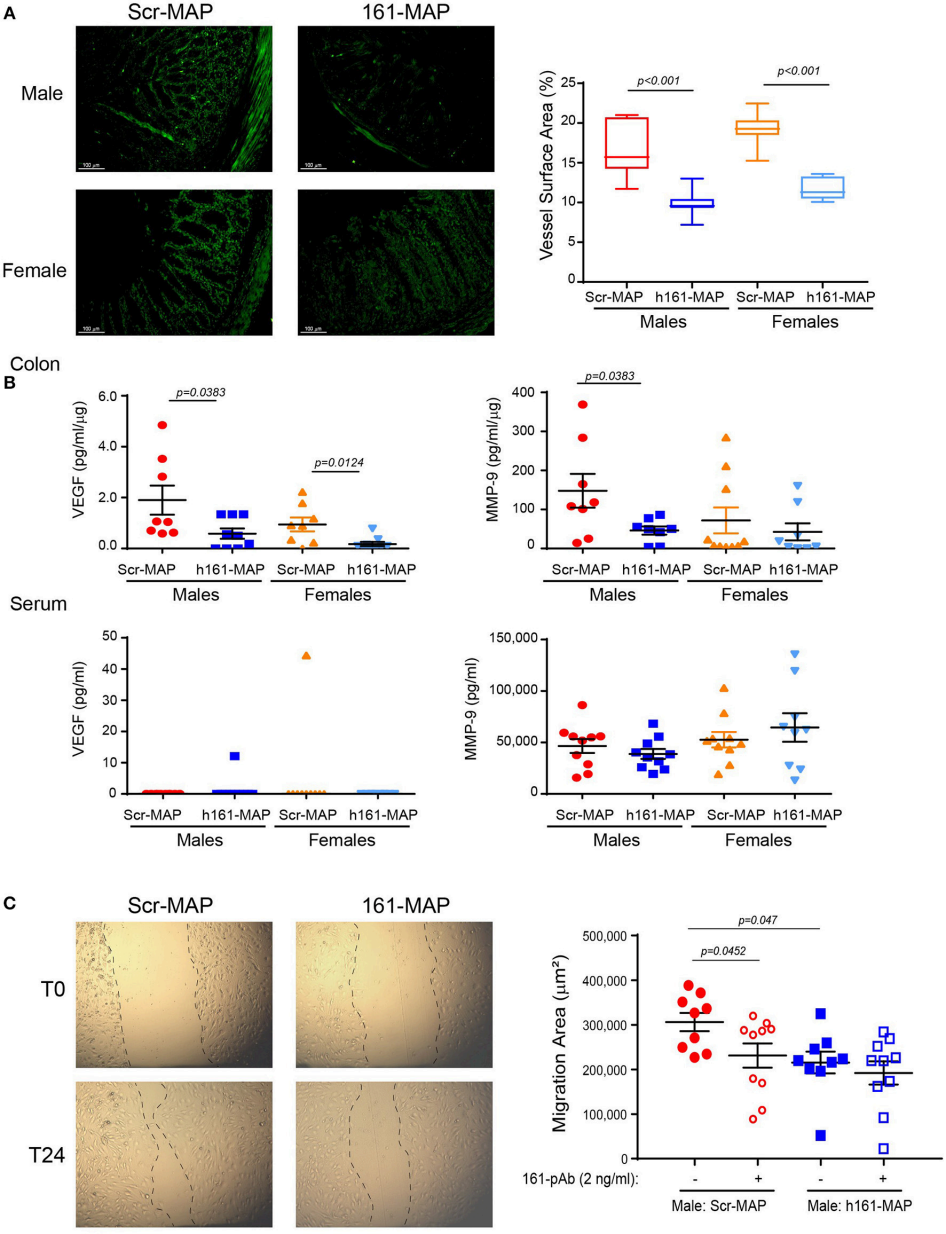
161-MAP active vaccination reduces angiogenesis. **(A)** Colon sections were stained for CD31 and the vessel surface area was calculated (*n* = 4 per group). Scale bar is 100 μm. **(B)** Concentrations of VEGF and MMP-9 were determined in serum sample by ELISA, and in the colon lysates, normalized to the total protein amounts (*n* = 9 per group). **(C)** Wound scratch assay: colon lysates (25 μg of total protein) were diluted (1:4) and applied onto a confluent layer of the mouse bEND3 endothelial cells (10^5^ cells/ 96-plate well) that was scratched with a toothpick. Images were acquired at the beginning of the experiment (T0) and at the end after 24h (T24). The migration area was calculated by subtracting the area of the wound at T24, after endothelial cell migrated and partially closed the wound, from the area of the wound at T0. An EMMPRIN specific blocking antibody (161-pAb) was added to some of the wells as indicated. (*n* = 9–10 for the male mice, *n* = 8 for the female mice). Magnification is x4.

Angiogenesis was evaluated by the microvessel density that was assessed by CD31 staining. In the control Scr-MAP vaccinated groups, blood vessels were abundant in the muscularis mucosa, and infiltrated into the LP (Figure 4A). In contrast, both male and female 161-MAP vaccinated mice exhibited reduced amount of blood vessels in both the muscularis mucosa and in the LP (by 1.6-fold, *p* < 0.001, 95% CI for male 4.5, 8.9], and for females [5.9, 9.2]. As expected, the reduction in EMMPRIN levels, a known inducer of VEGF and MMPs expression, led to a similar reduction in local VEGF levels in both male and female mice (by 3- and 5-fold, respectively, *p* < 0.05, 95% CI for male [0.08, 2.5] and for female [0.2, 1.35]), whereas in the serum VEGF was hardly detected in both genders for both 161-Scr-MAP and 161-MAP vaccinated mice (Figure 4B). However, colon MMP-9 levels were reduced only in the 161-MAP male vaccinated group (by 3-fold, *p* = 0.0383, 95% CI [6.3, 197.6]), but not in the female vaccinated group. Although the local reduction of EMMPRIN levels was reflected in the serum, MMP-9 serum levels and the lack of VEGF serum levels were unchanged by the 161-MAP vaccination.

To demonstrate the involvement of EMMPRIN in the angiogenic potential, we subjected the extracted proteins to bEND3 endothelial scratch assay. The ability of the lysates to trigger wound healing, that requires endothelial cell proliferation and migration, was assessed by measuring the area to which bEND3 cells had migrated, and the involvement of EMMPRIN was demonstrated by the neutralizing activity of the anti-EMMPRIN antibody (161-pAb). The baseline values of bEND3 cell migration, without addition of any protein extract, were similar and not different from the control Scr-MAP group (mean migration area 306,085 ± 20,493 mm). Relative to lysates obtained from the male Scr-MAP control group, the 161-MAP vaccinated male group reduced the migration of endothelial cells (by 1.42-fold, *p* < 0.047, 95% CI [23,426, 157,945], Figure 4C), and the addition of the antibody to male Scr-MAP lysates had a similar effect (*p* < 0.045). Likewise, the 161-MAP vaccination reduced migration in the female mice (1.6-fold, *p* < 0.019, 95% CI [133,284, 474,369]) (data not shown). However, addition of the antibody to the 161-MAP colon lysates in both male and female mice did not show any additional effect (Figure 4C), suggesting that the vaccination already neutralized EMMPRIN in these lysates.

### 161-MAP Vaccination Changes Immune Cell Infiltration, the Microenvironment, and Cell Viability

**Figure 5:**
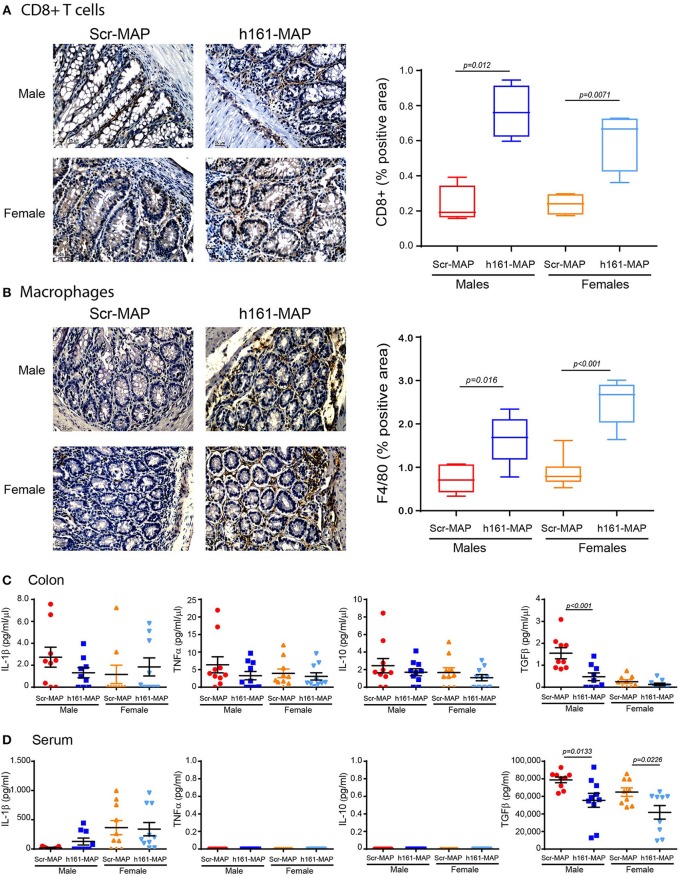
161-MAP active vaccination increases infiltration of CD8^+^ T cells and macrophages, and reduces TGFβ concentrations. Colon tissue sections were stained for **(A)** CD8^+^ or **(B)** for F4/80, and analyzed for their distribution (*n* = 4–5 in each group). Scale bar is 25 μm. **(C)** Proteins were extracted from colon segments and the concentrations of IL-1β, TNFα, IL-10 and TGFβ were determined in the lysates by ELISA and normalized to the total protein (*n* = 9–10). **(D)** Concentrations of the same cytokines were determined in the serum samples (*n* = 9–10).

As DSS-induced colitis is an inflammatory disease, and the vaccination process is likely to stimulate immune cells, we next stained colon sections for the presence of CD8^+^ T cells, macrophages and neutrophils. As expected, infiltration of CD8^+^ T cells, reflecting the stimulation of the adaptive immune system, was increased in the 161-MAP vaccinated mice in comparison to the control mice (by 3.3-fold, *p* = 0.012 for the males, 95% CI **[**0.3, 0.76], by 2.5-fold, *p* = 0.0071 for the females, 95% CI [0.14, 0.59] Figure 5A). Likewise, infiltration of macrophages was also increased (by 2.2-fold, *p* = 0.0016 for the males, 95% CI [0.41, 1.4], by 2.8-fold, *p* < 0.001 for the females, 95% CI [1.2, 2.1], Figure 5B). In contrast, neutrophil infiltration, that is characteristic of the innate immunity in acute inflammation, remained unchanged (data not shown).

To learn about the mode of activation of these cells, we next evaluated cytokine concentrations in serum samples and locally in the colon lysates. Concentrations of IL-1β, TNFα, and IL-10 in the serum and the colon were not different between the control and the 161-MAP vaccinated mice, in both males and females (Figures 5C,D), suggesting that the pro-inflammatory process was no longer active at this late stage. In contrast, levels of TGFβ were reduced in colon lysates of 161-MAP vaccinated male mice in comparison to the Scr-MAP vaccinated mice (by 3-fold, *p* = 0.03, 95% CI [0.4, 1.7]), whereas in female mice this trend (2-fold difference) did not reach significance. In the serum samples, TGFβ was reduced in both male and female 161-MAP vaccinated mice relative to their respective controls (by 1.4-fold and 1.5-fold, *p* < 0.05, 95% CI for male [4,357, 42,198] and for female [3,703, 42,412]). The high levels of TGFβ in the control Scr-MAP vaccinated mice together with no change in the low levels of the pro-inflammatory cytokines suggest that by day 15, the pro-inflammatory response was already replaced with a regeneration program. The relative reduction in those TGFβ levels in 161-MAP vaccinated mice suggests that a moderate repair program was in place.

**Figure 6:**
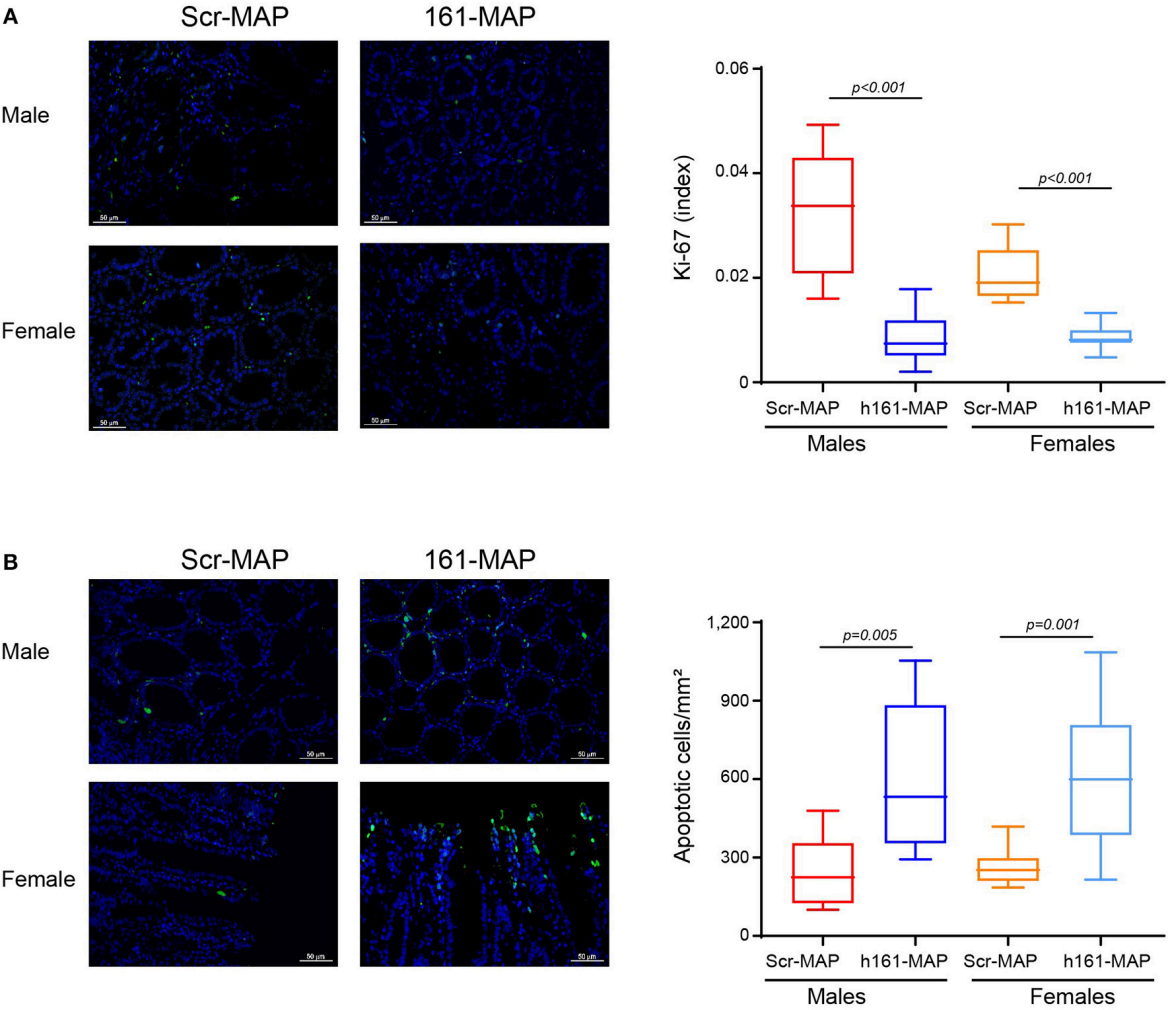
161-MAP active vaccination reduces proliferation and enhances apoptosis. Colon tissue sections were stained for Ki-67 or DNA strand breaks (TUNEL assay). **(A)** Representative images of Ki-67 staining (scale bar is 50 μm) and their quantitation (Green, proliferating cells; blue, DAPI staining of nuclei; white, merged co-localization, *n* = 3–4 in each group). **(B)** Representative images of the TUNEL assay and their quantitation. (Green, apoptotic cells; blue, DAPI staining of nuclei; white, merged co-localization, *n* = 3–4 in each group). Scale bar is 50 μm.

Staining the colon sections for Ki-67 revealed that proliferation in the 161-MAP vaccinated groups was reduced relative to the Scr-MAP controls (by 3.7-fold, *p* < 0.001 for males 95% CI [0.016, 0.03], and by 2.4-fold, *p* < 0.001 for females, 95% CI [0.009, 0.015], Figure 6A). In contrast, the number of apoptotic cells, as assessed by the TUNEL assay, was increased in the 161-MAP vaccinated mice relative to the Scr-MAP vaccinated controls (by 2.5-fold, *p* = 0.0005 for males, 95% CI [176.6, 544.6], by 2.3-fold, *p* = 0.0001 for females, 95% CI [191.2, 507.3], Figure 6B).

## Discussion

The mechanisms that drive UC and its analogous model of DSS-induced colitis are not fully understood, although increased ROS and pro-inflammatory cytokines, pathological angiogenesis, as well as the composition of the microbiota in the gut have been implicated (4, 6). In many of the treated patients, the drugs currently in use do not exert sufficient beneficial effects, suggesting that additional pathological mechanisms, that could potentially be targeted, are at play. Here we demonstrate that by selectively targeting EMMPRIN, a multifunctional protein that is primarily involved in angiogenesis, we reduced angiogenesis and ameliorated clinical manifestations of the DSS-induced colitis model, including weight loss and disease severity, pointing to the central role that angiogenesis plays in this model.

EMMPRIN is highly expressed in the colon, especially in the crypt base columnar cells. As the chaperon of the monocarboxylate transporter family (particularly of MCT-1 and MCT-4) it has an important function in the transport of monocarboxylate anions, such as lactate, pyruvate, ketone bodies and the short-chain fatty acids acetate, propionate and butyrate (20), all of which are particularly important in intestinal function. Additionally, EMMPRIN may help in recruiting leukocytes to the inflamed site, and support their adhesion to endothelial cells. Despite its important role, this is the first demonstration of the involvement of EMMPRIN in colitis, to the best of our knowledge. We show here that targeting EMMPRIN reduces angiogenesis, but the reduction in EMMPRIN expression in the 161-MAP vaccinated mice suggests that other functions of EMMPRIN may also be affected. These aspects deserve additional exploration that is outside the scope of the current work.

The vaccination reduced EMMPRIN expression and led to reduced microvessel density and angiogenesis. Angiogenesis is a necessary process, as it promotes and sustains inflammation by supplying nutrients, allowing increased leukocyte influx and promoting endothelial cell local production of chemokines, cytokines, and MMPs. In fact, many mediators exhibit a dual role as pro-angiogenic and pro-inflammatory, linking the two processes. For example, VEGF, which is a potent pro-angiogenic factor as well as a chemoattractant for macrophages, has been shown to increase mucosal angiogenesis, promote leukocyte adhesion and worsen the clinical outcome in both IBD patients and in a DSS-induced colitis model (7). Pathogenic angiogenesis and elevated levels of both VEGF and MMP-9 were demonstrated in different models of UC, including a DSS-induced colitis model (7, 21). Furthermore, VEGF has been implicated in increasing vascular permeability, which in the context of colitis may have an additional effect by allowing bacteria to invade into the LP, thus enhancing the inflammatory process (10, 22).

In the past, attempts have been made to target VEGF or its receptor VEGFR2. For example, targeting VEGFR2 with the monoclonal DC101 antibody in a DSS-induced colitis model did not inhibit angiogenesis or improve disease severity, probably due to VEGF-independent compensatory pathways that maintained downstream signaling events (23). The authors suggested that this monotherapy might have had better effects if used in combination with another monoclonal antibody that targeted another angiogenic mediator. This may have been the case in our EMMPRIN vaccination, as EMMPRIN is a mediator upstream of VEGF that also induces MMP-9. Indeed, we show a reduction (at least in the male groups) of both VEGF and MMP-9, two potent pro-angiogenic mediators. Thus, in contrast to the previous study, we succeeded in improving disease severity and in reducing angiogenesis, while demonstrating again the importance of VEGF in the DSS-induced colitis model.

Since inflammation and angiogenesis are interconnected processes, we expected that targeting angiogenesis through EMMPRIN vaccination would also reduce inflammation. For example, reduction of VEGF using anti-VEGF antibody has reduced vascular permeability and influx of immune cells into the colon in an experimental colitis model (22). EMMPRIN itself has a role in recruiting leukocytes, and therefore, targeting it was expected to reduce immune infiltrate. However, we show that 15 days after the onset of inflammation by the DSS, more macrophages and CD8^+^ T cells were present in the LP, and colonic levels of pro-inflammatory cytokines, such as TNFα and IL-1β, were unchanged by the vaccination. We suggest that these results can be explained by the duration of the model. Most studies, with or without interventions, follow a design where DSS is administered for 5–7 consecutive days and then the mice are immediately euthanized without a DSS-free period that allows regeneration and repair. Thus, the status of immune activation is measured at the peak of the innate inflammatory response. In our experimental design, the mice are allowed to drink DSS-free water after 5 days of exposure, giving them the chance to repair intestinal damage. In this kinetics, disease is most severe around day 9, 4 days after DSS is no longer administered. It might be argued that since we stimulated the adaptive immune system prior to DSS administration, it is possible that the innate and adaptive immune responses occur simultaneously, prolonging the time of maximal damage. However, since the irritation to the intestine was stopped after 5 days by supplying DSS-free drinking water, the mice have entered into a repair or healing stage. Thus, the intestinal milieu was probably reflecting a resolution state, rather than a pro-inflammatory response with high cytokine concentrations, even if the inflammatory cells were still physically present in the microenvironment. Supporting our premise are studies of DSS-induced colonic tissue that show reduced cytokine production at the mRNA or protein levels in the resolution phase compared to the acute phase of inflammation (24–26).

Several evidences support our conclusion that the immune response is at the repair stage. First, the low number of infiltrating neutrophils in the colon samples did not change upon vaccination, suggesting that the system was no longer in acute inflammation. In contrast, during the acute phase of DSS-induced colitis model the innate immune cells, especially neutrophils and macrophages, massively infiltrate the LP, and elevated levels of the pro-inflammatory cytokines they secrete, such as TNFα, IL-1β, and IL-17 are observed (27). However, upon removal of DSS, the acute response gradually changes into a chronic response, pro-inflammatory cytokines are decreased, and Th2 cytokine levels are increased (1, 2). In particular, IL-4 and TGFβ have been shown to be critical for regeneration of intestinal epithelial cells (28, 29). Comparing vaccinated and control mice, we found no change in the amount of infiltrating neutrophils, but CD8^+^ T cells and macrophages were increased, suggesting that the adaptive immune response, rather than the innate immunity, was specifically increased in the 161-MAP vaccinated mice. The levels of TNFα, IL-1β, and IL-10 were low and showed no difference between the groups, whereas TGFβ levels were reduced compared to the Scr-MAP vaccinated control mice. This is consistent with the importance of this cytokine in tissue regeneration, and suggests that the damage in the vaccinated mice during the early stages was relatively reduced, leading to a reduced need for regeneration. Furthermore, turnover of the surface epithelium in the colon takes about 5–8 days. Although signs of inflammation are clearly visible in the vaccinated mice, they also demonstrate restoration of the crypts and epithelium, suggesting reduced inflammation and damage. Alternatively, we surmise that the 161-MAP vaccination triggered an early EMMPRIN-specific pro-inflammatory response, allowing for DSS-damaged epithelial cells that express EMMPRIN to be cleared faster, and helping to promote a rapid regeneration, which was reflected by the reduced loss of weight and disease activity scores.

DSS is believed to directly kill intestinal epithelial cells, cause barrier dysfunction and induce innate immunity, all leading to enhanced epithelial injury during acute inflammation (3, 4, 30). Studies show that relative to control mice without colitis, DSS-induced colitis increases apoptosis and decreases cell proliferation at the early stages of acute colitis, thus contributing to the barrier dysfunction (31). However, once DSS is removed, the intestine begins to proliferate, in order to regenerate the epithelial layer and restore epithelial barrier function. Increased proliferation underlies an attempt to regenerate the epithelial layer and restore barrier functions (3, 5). Thus, we would expect increased proliferation and reduced apoptosis during the regeneration phase in the 161-MAP group. In contrast, we demonstrate reduced proliferation and enhanced apoptosis in the 161-MAP vaccinated mice relative to the control Scr-MAP vaccinated mice. We suggest that the high proliferation in the Scr-MAP groups reflects an ongoing regeneration at this time point (10 days after cessation of DSS administration), whereas the relatively reduced proliferation observed in the 161-MAP vaccinated groups indicates earlier recovery and a reduced need for regeneration at this time. Likewise, the enhanced apoptosis observed in the 161-MAP vaccinated groups may reflect the death of both epithelial and non-epithelial cells, for example neutrophils, which typically appears at the end of the regeneration phase. As apoptotic neutrophils have been shown to shift macrophage activation toward a healing phenotype (32), and based on the improvement in the DAI and weight loss in the 161-MAP vaccinated groups, we propose that the increase in apoptotic cells may in fact protect the colon and help reduce inflammation.

The protective effects of the 161-MAP vaccination in the DSS-induced colitis model are comparable to our recent results with the same vaccine in implanted and metastatic tumor models, where we used the CT26 colon carcinoma cells among others. We demonstrated there that relative to Scr-MAP vaccinated mice, tumors or metastases were reduced and even eliminated, angiogenesis and its mediators VEGF and MMP-9 were reduced, more CD8^+^ T cells and macrophages infiltrated the tumors and were engaged in killing tumor cells, TGFβ was reduced and an increase in a Th1/M1 gene signature was detected (33). However, tumor models represent an ongoing chronic inflammation, with continuous exposure to inflammation-inciting triggers and mediators. In contrast, in our design of the DSS-induced colitis model, we allowed enough time for regeneration following cessation of DSS administration. To delineate the full spectrum of the protective effects of the 161-MAP vaccination in the DSS-induced model, a follow-up study looking at multiple time points during the dynamic healing process should be conducted, and immune cells should be isolated from the LP to phenotype and characterize their exact mode of activation.

Many autoimmune diseases are known for their gender bias, generating our interest to examine this phenomenon in our DSS-induced colitis model. Indeed, we observed that despite the basic similarities in kinetics, the female group generally exhibited a more moderate inflammation, reflected by less severe weight loss and DAI scores. This is in agreement with other studies that found that male mice respond faster and develop a more significant and aggressive colitis relative to female mice when exposed to DSS (3), and that STAT-1 deficiency or IRAK-1 deficiency render male, but not female, mice more resistant to DSS-induced colitis (34, 35). However, not all parameters were consistent with this trend, as the histology score and the colon length were similar between males and females. Other studies that showed difference in weight loss but no difference in colon length between the genders attributed these findings to mild colitis being induced (35, 36).

In summary, we show that the critical component of angiogenesis can be targeted in DSS-induced colitis, by vaccinating against the pro-angiogenic mediator EMMPRIN protein. This vaccination improved disease severity, reduced angiogenesis and expedited regeneration, although a direct effect on inflammatory cytokines was not observed.

## Materials and Methods

### Experimental Mouse Model, Vaccination and Disease Activity Index (DAI)

C57BL/6J OlaHsd male and female mice (8 weeks old, Envigo Laboratories, Jerusalem, Israel), were housed in specific pathogen free (SPF) conditions and kept with a 12 h light/dark cycle and access to food and water *ad libitum*. To vaccinate mice we used the synthetic multiple antigenic peptide (MAP) derived from the human sequence of the EMMPRIN protein (sequence: GHRWLKGGVVLC, designated h161-MAP), or a peptide with the same amino acids in a scrambled order used as a negative control (sequence: WCRGGGLKMRVH, designated Scr-MAP). Peptides were synthesized by the standard stepwise solid-phase procedure using Fmoc chemistry on β-Ala-Wang resin, conjugating the peptides onto an octa-branched lysine core (Yuan Yu Bio-Teck), and purity was confirmed by HPLC and mass spectroscopy. Using the human sequence, rather than the mouse sequence, reflected the homology between the two sequences, and was shown before to trigger an equally effective EMMPRIN-specific response (33). For the first vaccine injection, the 161-MAP and Scr-MAP (50 μg each) were emulsified in complete Freund's adjuvant (CFA) for the first vaccine injection, and additional three vaccination injections where the same amount of MAPs was emulsified in incomplete Freund's adjuvant (IFA), were administered subcutaneously to each mouse every 7 days. Four days after the last boost injection, colitis was induced with 2% DSS (MW 36–50 kDa, cat. No. 160110, MP Biomedicals, LLC Solon, OH) administered in the drinking water for five consecutive days, after which their drinking water were replaced with regular water, and the mice were left for additional 10 days. At the end of the experiment, mice were euthanized and their colon tissue and serum were harvested for later analyses. Disease activity index (DAI) was calculated as the average of loss of weight, stool consistency and bleeding and evaluated daily for each mouse. Change in weight relative to the weight of each mouse on the first day of DSS administration was given the scores: 0, if < 1%; 1, 1–5%, 2, 5–10%, 3, 10–15%; 4, >15%; Consistency of the stool was assigned the scores: 0, normal stool; 2, loose or pasty pellets; 4, diarrhea. Presence of occult blood (measured with Hemooccult, SENSA, Beckman Coulter, Brea, CA) was given the following scores: 0, normal; 2, positive occult blood; 4, rectal bleeding.

### Histology and Scoring

Colon sections were fixed in 4% formalin and paraffin embedded, and then 4 μM sections were stained with hematoxylin and eosin (H&E). Histological scoring, assessing the severity of the model, was based on four parameters. Epithelial loss was scored as follows: 0, no epithelial loss; 1, loss of up to 5% of the epithelial surface; 2, loss of 5–10% of the epithelial surface; 3, loss of >10% of the epithelial surface. Crypt integrity was evaluated as follows: 0, Intact crypt; 1, loss of 0–10% of the crypts; 2, loss of 10–20% of the crypts, 3, loss of >20% of the crypts. Inflammatory infiltrate was assigned the following score: 0, no infiltration; 1, mild leukocyte infiltrate; 2, moderate leukocyte infiltrate; 3, severe leukocyte infiltrate. Depletion of Goblet cells was estimated as: 0, no depletion of Goblet cells; 1, mild depletion of Goblet cells; 2, moderate depletion of Goblet cells; 3, severe depletion of Goblet cells.

### Immunohistochemistry, Immunofluorescence, and Immune Reactive Score

Four-micron thick paraffin embedded tissue sections were deparaffinized on a glass slide with xylene substitute K-Clear Plus (Kaltex) and rehydrated with decreasing ethanol immersions. Antigen retrieval for Ki-67 and F4/80 was performed by microwave heating in citrate buffer pH 6.0, for CD31 by immersing the slides in 42 mg/mL Proteinase XXIV solution (Sigma-Aldrich, Rehovot, Israel) for 10 min at 37°C, or in 20 mg/mL of Proteinase K in Tris buffer, pH 8.0 for the TUNEL kit. Endogenous peroxidase was quenched in 3% H_2_O_2_ solution for 10 min, slides were blocked with 5% BSA and incubated overnight at 4°C with the following primary antibodies: rat anti-mouse EMMPRIN (R&D systems, MAB772, Minneapolis, MN, USA) diluted 1:250; rat monoclonal anti-F4/80 (Abcam, ab6640, Cambridge, UK) diluted 1:200; rabbit polyclonal anti-CD8 (Bioss, bs-0648R, Woburn, MA, USA) diluted 1:400. After washing, the antibodies were detected with HRP-Polymer anti-rabbit (Zytomed, Berlin, Germany) or with the N-Histofine Simple Stain Mouse MAX PO (Rat) (Nichirei Bioscience, Tokyo, Japan) for 1 h and the DAB substrate Kit (Zytomed systems). All sections were counterstained with hematoxylin (Sigma) and coverslips were applied using Pertex mounting medium (Histo-lab Products AB). For the CD31 and Ki-67, we used the primary antibodies rat monoclonal anti-CD31 (Acris Antibodies, BM4086, Herford, Germany) diluted 1:50 and rabbit monoclonal anti-Ki67 (Abcam, ab16667) diluted 1/140. Secondary antibodies were donkey Alexa Fluor 488-conjugated anti-rat IgG (Abcam, ab150153), or donkey Alexa Fluor 488-conjugated anti-rabbit (Abcam, ab150061), respectively, diluted 1:500. Nuclei were stained with 300 nM DAPI (MP Biochemicals, LLC Solon, OH) and coverslips were applied using the fluorescent mounting medium (Agilent Dako, Carpinteria, CA). The TUNEL staining was performed using the *in situ* death detection kit POD (Roche Life Science, Mannheim, Germany) according to manufacturer's instructions. All sections were viewed under the bright field trinocular microscope (Olympus BX-60, Tokyo, Japan) and images were acquired with the MS60 camera and the MShot Image Analysis System V1 (MSHOT, Guangzhou Micro-shot Technology Co., Guangzhou, China). Vessel densities were assessed in CD31 stained sections by using a Weibel grid to calculate vessel surface area (37), and the fraction of Ki-67-positive tumor cells was calculated by the digital image analysis web application ImageJS (38). EMMPRIN expression was assessed using the modified H-score, which assigns an immune reactive score on a continuous scale of 0–300, based on the percentage of positive cells expressing the protein at different intensities. Staining was divided into three categories: 1 for “light staining,” 2 for “intermediate staining,” and 3 for “strong staining.” The percentage of positive cells was determined according to the positive surface area of cells measured with ImagePro plus 4.5 software, and the score was calculated using the formula: 1 × (%1 positive cells) + 2 × (%2 positive cells) + 3 × (%3 positive cells).

### Sandwich ELISA

The mouse cytokines were determined using ELISA kits (R&D systems,) according to the manufacturer's instructions. Serum samples were diluted 1:4 (for IL-1β, IL-10, and TNF) or 1:100 (for TGFβ, MMP-9, and VEGF), and tissue lysate samples were normalized to the total protein. Serum EMMPRIN concentrations were measured with an ELISA kit (Abcam, ab215405) at a dilution of 1:200, according to the manufacturer's instructions, or normalized to total protein in tissue lysates.

### *In vitro* Wound Scratch Assay

*In vitro* wound scratch assay was performed as described before (17), with the mouse bEND3 endothelial cell monolayers (10^5^ cells) seeded in 96-well dishes and incubated with 25 mg of total protein extracted from colon samples of the control or 161-MAP vaccinated mice groups. To demonstrate EMMPRIN involvement, we added the rabbit anti-mouse EMMPRIN polyclonal antibody (161-pAb, 2 ng/ml) that we previously produced (19), to some of the wells. Images of the field of injury were acquired at the beginning of the experiment (T0) and after 24 h (T24) using the ImagePro plus 4.5 software (Media Cybernetics, Inc., Rockville, MD, USA), and the wound area was measured at both times. The migration area, reflecting the area to which endothelial cells migrated in order to close the wound, was calculated by the subtraction of the area at T24 from the area at T0.

### Statistical Analyses

All values are presented as means ± SE and all comparisons are presented with the 95% CI for the difference between the means. Significance between two groups was determined using the two-tailed unpaired *t*-test. Differences between experimental groups accounting for time and treatment were analyzed using two-way analysis of variance (ANOVA) and the *post hoc* Bonferroni's multiple comparison test. *P*-values exceeding 0.05 were not considered significant.

## Ethics Statement

Mice were cared for in accordance with the procedures outlined in the NIH Guideline for the Care and Use of laboratory Animals, and all experiments were performed under the approved protocol (IL-0350315) issued by the Animal Care and Use Committee of the Technion-Israel Institute of Technology.

## Authors Contributions

ES performed the animal experiments and carried out all ELISA analyses, as well as wound assays. VB was in charge of the immunohistochemical staining. MAR designed the study, analyzed and interpreted the results, and wrote the manuscript.

### Conflict of Interest Statement

ES and VB declare that the research was conducted in the absence of any commercial or financial relationships that could be construed as a potential conflict of interest. MR is an inventor of a patent (US Grant US9688732B2, EP application EP2833900A4) related to the research described in the manuscript.
